# *In vitro* effect of two commercial anti-coccidial drugs against myxospores of *Kudoa septempunctata* genotype ST3 (Myxozoa, Multivalvulida)

**DOI:** 10.1051/parasite/2017012

**Published:** 2017-03-21

**Authors:** Meejung Ahn, Seunghwan Won, Bongjo Kang, Po Gong, Eunho Yoo, Subramanian Dharaneedharan, Yeounghwan Jang

**Affiliations:** 1 School of Medicine, Jeju National University Jeju 63243 Republic of Korea; 2 Ocean and Fisheries Research Institute, Jeju Special Self-Governing Province, Pyoseon-myeon, Segwipo-si Jeju 63629 Republic of Korea; 3 Marine Applied Microbes and Aquatic Organism Disease Control Laboratory, Department of Aquatic Biomedical Sciences, School of Marine Biomedical Sciences, Jeju National University Jeju 690-756 Republic of Korea

**Keywords:** Anti-coccidial drug, *Kudoa septempunctata*, ST3 genotype, Foodborne disease, Amprolium hydrochloride, *Paralichthys olivaceus*

## Abstract

*Kudoa septempunctata* (Myxozoa: Multivalvulida) myxospores infect the trunk muscles of olive flounder (*Paralichthys olivaceus*). In this study, two popular commercially formulated anti-coccidial drugs (amprolium hydrochloride and toltrazuril) were serially diluted and incubated with purified mature *Kudoa septempunctata* myxospores. The viability of *K. septempunctata* spores was determined after a 2-day incubation followed by Hoechst 33342 and propidium iodide staining, and scanning electron microscopy. Amprolium hydrochloride significantly decreased spore viability (18% of control) at a concentration of 920 μg/mL, whereas toltrazuril showed almost no effect (83% of control). Viability of the control (untreated spores) was 90%. *In vivo* studies are required to confirm the efficacy of amprolium hydrochloride in fish infected with *K. septempunctata* myxospores on their growth and immune system performance.

## Abstract (fr)

Les myxospores de *Kudoa septempunctata* (Myxozoa, Multivalvulida) infectent les muscles du cardeau hirame (*Paralichthys olivaceus*). Dans cette étude, deux médicaments anticoccidiens commercialisés populaires (chlorhydrate d’amprolium et toltrazuril) ont été dilués en série et incubés avec des myxospores mûres purifiées de *K. septempunctata*. La viabilité des spores de *K. septempunctata* a été déterminée après une incubation de 2 jours, suivie par des colorations au Hoechst 33342 et à l’iodure de propidium et microscopie électronique à balayage. Le chlorhydrate d’amprolium a significativement diminué la viabilité des spores (18 % du témoin) à une concentration de 920 μg/mL, alors que le toltrazuril n’a pratiquement pas eu d’effet (83 % du témoin). La viabilité du témoin (spores non traitées) était de 90 %. Des études *in vivo* sont nécessaires pour confirmer l’efficacité du chlorhydrate d’amprolium chez les poissons infectés par les myxospores de *K. septempunctata* sur leur croissance et leur performance immunitaire.

## Introduction

Global fish production for human consumption has outpaced population growth in the past five decades, with preliminary estimates of annual per capita intake of >20 kg, which is double the level in the 1960s, due to advancements in aquaculture [18]. In particular, olive flounder, *Paralichthys olivaceus*, is one of Korea’s most commercially important marine aquaculture species with annual production of 45,758 metric tons and was ranked first among Korean marine culture fish in 2015 [11].

The myxosporean parasite *Kudoa septempunctata* was first identified in the trunk muscle of cultured olive flounder imported from Korea to Japan in 2010 [12]. Mature *Kudoa septempunctata* spores contain six or seven shell valves and polar capsules [12] and are genetically classified into three groups: ST1, ST2 and ST3 [17]. The ST1 and ST2 genotypes were isolated from wild and farmed flounder fish in Japan, whereas the ST3 genotype was imported to Japan from Korea [17].

The World Health Organization cautions that eating raw fish could cause illness due to the presence of foodborne microorganisms [19]. *K. septempunctata* has recently been linked to human food poisoning cases in Japan [15]. *K. septempunctata* spores have been isolated from raw olive flounder within 20 h after the fish was consumed by patients who developed diarrhoea, and the patients recovered completely within 24 h, suggesting that *K. septempunctata* spores are associated with transient clinical symptoms [10]. Several studies have reported clinical symptoms in animal models including the mouse [1, 7, 10], and permeability increase in the human intestinal epithelial monolayer [14]. Few reports on detection methods for *K. septempunctata* are available [10, 15, 16]. Contaminated fish occasionally reach the consumer through raw olive flounder fillets as infection is not tested for prior to consumption. The *K. septempunctata* life cycle within and outside the olive flounder has not been elucidated, and the microbe likely uses an annelid as an alternate host [20].

Prevention and control of myxosporean infections are essential but particularly difficult due to the lack of effective treatments and scarce knowledge on the transmission and life cycles of these marine parasites. Chemotherapy against myxosporean diseases is not well established, but therapeutics, such as toltrazuril and amprolium, have been reported efficacious against several myxosporean infections [3, 13].

Hence, the present study aims to evaluate the efficacy of two anti-coccidial agents used in poultry coccidiosis against purified myxospores. The anti-coccidial compounds were tested for the first time to assess their efficacy against *K. septempunctata* spores in an *in vitro* drug sensitivity assay. The findings presented in this report provide a foundation for further investigations to assess the *in vivo* effect of anti-coccidial drugs in fish infected with *K. septempunctata* myxospores on their physiological, biochemical and immune status, which could potentially be applied in flounder fish disease management.

## Material and methods

### Preparation of *K. septempunctata* spores

*Kudoa septempunctata*-infected olive flounder were collected from commercial fish farms located on Jeju Island, South Korea. The fish were thoroughly inspected for *Kudoa* myxospores under a microscope at 400× magnification and *K. septempunctata* was confirmed using real-time polymerase chain reaction (PCR) [10]. Heavy infections were defined as >10^5^ spores/g tissue. Heavily infected fish were filleted to purify the spores following Chase et al. [6]. Briefly, about 2 g of muscle tissue was ground in 10 mL of phosphate-buffered saline (PBS). The tissue extract was passed through 100- and 50-μm filters to remove debris and centrifuged at 1500 × *g* for 15 min at 4 °C. The pellet, which potentially contained *K. septempunctata* spores, was suspended in 1 mL of PBS, and the spores were counted using a haemocytometer and further purified using Percoll density gradient centrifugation [6]. The spore genotypes were assessed by conventional PCR to amplify the cytochrome *c* oxidase subunit I (*cox 1*; 751 bp) and large subunit rRNA (*rnl*; 817 bp) of the *K. septempunctata* mitochondrial gene [17]. The primer sets used were *cox 1*-F1 (5′-TTTGTTCATCGGCACAATTC-3′), *cox 1*-R1 (5′-ATAGCCTGGAACAAGGAATC-3′), *rnl*-F1 (5′-TGCCGTCATTCTGTTGTATT-3′), and *rnl*-R1 (5′-AATACCCATGCTGTGTTCAT-3′), as described in previous reports [1, 7, 17]. Negative controls (without template DNA) were included to check for contamination. The PCR products were sequenced on an ABI 3730XL DNA analyser (Applied Biosystems, Foster City, CA, USA). The mitochondrial genes were subjected to multiple sequence alignment using ClustalW (http://www.clustal.org) with MEGA v. 5.1 software.

### Viability assay by fluorescence microscopy

*Kudoa* spores were stained with Hoechst 33342 dye (5 mg/mL solution in water) (Invitrogen, Carlsbad, CA, USA) and propidium iodide (PI) (1 mg/mL solution in water) (Invitrogen) as modified by Yokoyama et al. [21, 22]. The fluorescent dyes were bound to DNA, and the chromatin in live spores was distinguished from that in dead cells. Live spores with intact cellular membranes do not stain with PI, while dead spores with defective membranes take up stain. On the other hand, Hoechst 33342 stains both live and dead spores. The drug-treated spores were rinsed in cold PBS and adjusted to 1 × 10^6^ spores/mL using a haemocytometer in a 1-mL volume. A 1-μL aliquot of Hoechst 33342 and PI stock solution was added to each 1 mL of spore suspension. The mixture was incubated for 10–20 min at room temperature. The immunofluorescence-stained spores were examined under a fluorescence microscope (BX-51; Olympus, Tokyo, Japan) and a standard microscope (ProgRes C7; Jenoptik, Jena, Germany). Spore viability was confirmed on 100 spores; bright blue-fluorescent Hoechst 33342 spores had normal chromatin and were live cells, whereas dead cells showed the red fluorescence of PI [22].

### Scanning electron microscopy (SEM)

Untreated and treated spores were fixed in 2.5% (v/v) glutaraldehyde at room temperature for 2 h, washed in PBS (pH 7.4), treated with 1% osmium tetroxide (OsO_4_) and then with distilled water for 1 h and dehydrated through a gradient ethanol series (40, 50, 60, 70, 80, 90 and 100%). The spores were treated with isoamyl acetate and 100% alcohol at ratios of 3:1, 2:2 and 1:3 and pure isoamyl acetate. Then, the samples were dried to critical point and covered in gold platinum. Finally, the samples were observed and photographed by SEM using a SUPRA 55VP (Carl Zeiss, Ltd., Cambridge, UK), with an acceleration voltage of 3 kV.

### Susceptibility assays

The *in vitro* activity of the anti-coccidial drugs was assessed after *K. septempunctata* (10^6^ spores/mL) were exposed for 48 h at 25 °C. Twofold serial dilutions of Ampros (9.6% amprolium hydrochloride; KBNP, Seoul, Korea) and Tolcoxin (2.5% toltrazuril; KBNP) were used, with the concentration ranging from 28.75 to 920 μg/mL. The experiments were performed separately for 24 and 48 h. We added 100 μL of stock *K. septempunctata* suspension to 900 μL of each drug concentration in 1.5-mL microcentrifuge tubes. The samples were vortexed and incubated for 24 and 48 h at 25 °C and were counted under a fluorescence microscope. The control contained no drugs and the minimum inhibitory concentration (MIC) was defined as the lowest concentration at which no distinct viability was detectable microscopically under normal light and fluorescent light microscopes. Each drug was tested three times.

### Statistical analyses

The means ± standard deviations of the assayed parameters were calculated for each group. The two-sample Student’s *t*-test was used to compare values between individual experimental and control groups. Differences were considered significant at *p* < 0.05.

## Results and discussion

**Figure 1.**
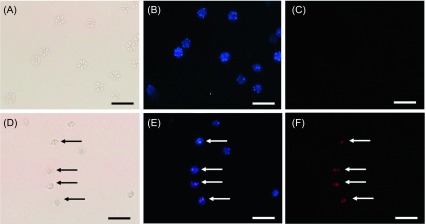
*Kudoa septempunctata* spores observed with normal light (A, D) and fluorescent light (B, C, E, F) and stained with Hoechst 33342 (blue fluorescence: B, E) and propidium iodide (red fluorescence: C, F). A–C, control (live spores, untreated); D–F, Ampros-treated spores. Arrows indicate dead spores. Scale, 20 μm.

**Figure 2.**
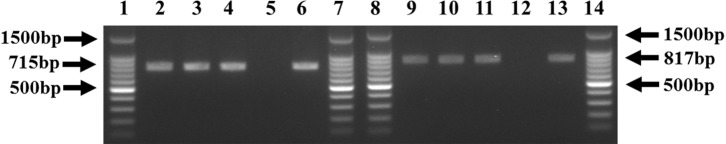
PCR amplification of the mitochondrial gene fragments from *Kudoa septempunctata* spores (in triplicate). Lanes: 1; SiZer^TM^-100 bp DNA Marker (iNtRON, Korea), 2–4; *cox 1* gene, 5; negative control, 6; positive control, 7; Size marker, 8; Size marker, 9–11; *rnl* gene, 12; negative control, 13; positive control, 14; Size marker.

Microscopic analyses of *K. septempunctata* isolated from infected olive flounder muscle tissues collected from fish farms on Jeju Island confirmed that purified *Kudoa* myxospores contain either six or seven shell valves and one polar capsule per spore (Fig. 1A), which was consistent with previous reports [1, 7]. Control spores with nuclei exhibiting only blue fluorescence (Hoechst 33342 positive and PI negative) were considered viable (Figs. 1B and 1C), whereas Ampros-treated spores with even just one nucleus exhibiting red fluorescence (Hoechst 33342 positive and PI positive) were considered dead (Figs. 1E and 1F). PCR analysis of the two mitochondrial genes *cox 1* and *rnl* from *K. septempunctata* resulted in amplification of 751-bp and 817-bp fragments, respectively (Fig. 2). The gene sequences obtained showed 100% similarity with those of *cox1*-3 (KU163620) and *rnl*-2 (KU163621) belonging to the ST3 genotype [1, 7].

Double staining with Hoechst 33342 and PI has been used extensively to assess cell viability, as it accurately discriminates between live and dead cells [4]. This vital stain assay was used to evaluate the survival of certain developmental stages of *Enteromyxum leei* in seawater [21]. Recently, Yokoyama et al. [22] evaluated the efficacy of temperature and chemical treatment for inactivating *K. septempunctata* spores *in vitro* using a double staining assay with Hoechst 33342 and PI.

**Figure 3.**
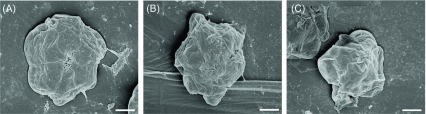
Scanning electron microphotographs of *Kudoa septempunctata* spores. (A) Untreated control; (B and C) incubated with Ampros after 48 h. Spores decreased in size and the shell valves became unstructured (B, C). Scales, 2 μm.

**Figure 4.**
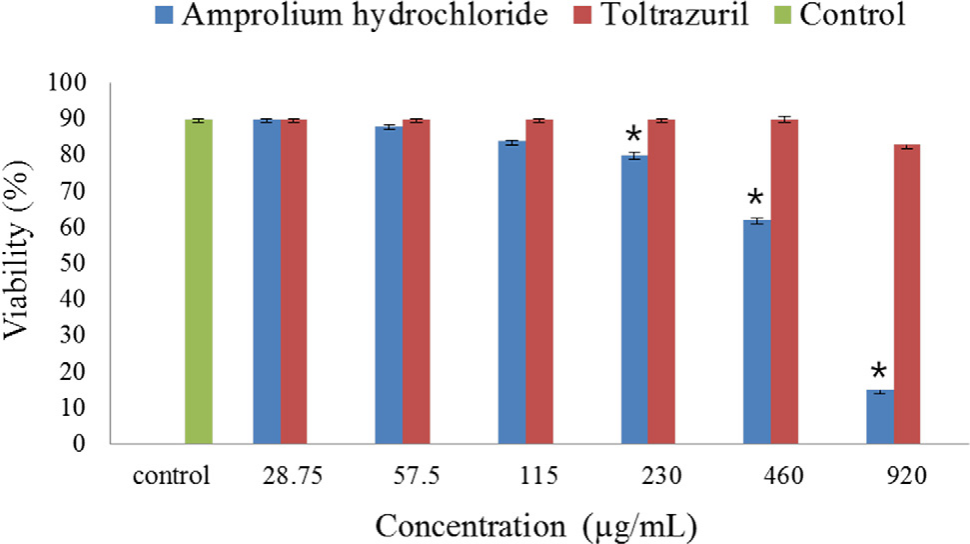
Viability of *Kudoa septempunctata* spores treated with different concentrations of amprolium hydrochloride and toltrazuril for 48 h as determined by fluorescent dye stains. Untreated spores were the negative control. Data are mean ± standard deviation. *Value shows significant difference from the control (*p* < 0.05).

In the present study, the *in vitro* effect of two anti-coccidial drugs, namely Ampros and Tolcoxin, on purified *K. septempunctata* spores was evaluated. Ampros showed a significant (*p* < 0.05) anti-parasitic effect by shrinking the size of spores from 8.23 ± 0.5 μm (control) to 6.89 ± 0.3 μm (treated), and the spore structure was distorted (Fig. 3). Matsukane et al. [12] reported a mean *Kudoa* spore size of 8.5 μm, determined by SEM analysis. *Kudoa* spores treated with Ampros exhibited 82% viability relative to the control (90%) at an MIC of 57.5 μg/mL, decreasing to 18% viability at an MIC of 920 μg/mL after the 48-h incubation, compared with that of untreated control spores (Fig. 4). However, Tolcoxin-treated *Kudoa* spores showed 83% viability relative to the control even at the highest test concentration (920 μg/mL). Additionally, the present study targeted only mature spores and not other developmental stages. Thus, the drugs’ efficacies may be higher when applied *in vivo* to infected fish at a range of developmental stages, of which the presporogonic ones are likely to be more susceptible than are mature spores [13]. Amprolium is a structural analogue of thiamine (vitamin B1) that competitively inhibits thiamine use by the parasite. It has been considered low to moderately toxic to different aquatic organisms [5] and the combination of amprolium with salinomycin is effective without negative effects on body weight or histopathological evidence of toxicity in some marine fish [9]. Moreover, dietary inclusion of several anti-coccidial drugs, including clamoxyquin, proguanil and fumagillin, has been found effective against myxozoan infections in finfish [2, 3]. Specifically, nicarbazin, an anti-coccidial drug, has been used against trunk muscle infection of *K. thyrsites* in Atlantic salmon smolts [8].

Hence, this study reports that among the two tested anti-coccidial drugs, amprolium hydrochloride could be potentially useful as an anti-parasitic drug with an MIC value of 57.5 μg/mL. Further *in vivo* studies are required to assess the positive and negative effects of these anti-coccidial drugs in host fish, such as *P. olivaceus.*

## Conflict of interest

The authors declare that they have no conflict of interest.
